# A Wearable Goggle Navigation System for Dual-Mode Optical and Ultrasound Localization of Suspicious Lesions: Validation Studies Using Tissue-Simulating Phantoms and an Ex Vivo Human Breast Tissue Model

**DOI:** 10.1371/journal.pone.0157854

**Published:** 2016-07-01

**Authors:** Zeshu Zhang, Jing Pei, Dong Wang, Qi Gan, Jian Ye, Jian Yue, Benzhong Wang, Stephen P. Povoski, Edward W. Martin, Charles L. Hitchcock, Alper Yilmaz, Michael F. Tweedle, Pengfei Shao, Ronald X. Xu

**Affiliations:** 1 School of Engineering Science, University of Science and Technology of China, Hefei, China; 2 Department of Surgery, Anhui Medical University, Hefei, China; 3 College of Engineering, The Ohio State University, Columbus, Ohio, United States of America; 4 Division of Surgical Oncology, Department of Surgery, The Ohio State University Wexner Medical Center, Columbus, Ohio, United States of America; 5 Pathology Department, College of Medicine, The Ohio State University, Columbus, Ohio, United States of America; 6 Radiology Department, Wright Center for Innovation, College of Medicine, The Ohio State University, Columbus, Ohio, United States of America; AntiCancer Inc., UNITED STATES

## Abstract

Surgical resection remains the primary curative treatment for many early-stage cancers, including breast cancer. The development of intraoperative guidance systems for identifying all sites of disease and improving the likelihood of complete surgical resection is an area of active ongoing research, as this can lead to a decrease in the need of subsequent additional surgical procedures. We develop a wearable goggle navigation system for dual-mode optical and ultrasound imaging of suspicious lesions. The system consists of a light source module, a monochromatic CCD camera, an ultrasound system, a Google Glass, and a host computer. It is tested in tissue-simulating phantoms and an ex vivo human breast tissue model. Our experiments demonstrate that the surgical navigation system provides useful guidance for localization and core needle biopsy of simulated tumor within the tissue-simulating phantom, as well as a core needle biopsy and subsequent excision of Indocyanine Green (ICG)—fluorescing sentinel lymph nodes. Our experiments support the contention that this wearable goggle navigation system can be potentially very useful and fully integrated by the surgeon for optimizing many aspects of oncologic surgery. Further engineering optimization and additional in vivo clinical validation work is necessary before such a surgical navigation system can be fully realized in the everyday clinical setting.

## 1. Background

Surgical resection remains the primary curative treatment for many early-stage cancers, including breast cancer. However, the major challenges facing surgeons in the operating room during cancer surgery are the correct identification of all sites of disease, the accomplishment of complete surgical resection, and accurate assessment of the surgical resection margins [1]. Incomplete surgical resection during cancer surgery can lead to the need of subsequent additional surgical procedures, can result in increased patient anxiety and stress, and can delay the initiation of subsequent necessary postoperative adjuvant therapies [2]. Specifically related to breast cancer, surgical resection margin positivity with breast conserving surgery has been reported in a wide range from 6% to 60%, with most series reporting in the range from 15% to 30% [3–5]. Permanent histopathologic analysis, using hematoxylin and eosin (H&E), remains the current gold standard for the microscopic assessment of surgical resection margins [6]. However, this process is labor-intensive, is not easily accomplished in real-time, and realistically only assesses a minute fraction of both the entire margin surface area and the entire 3-dimensional volume of the surgical resection specimen [7, 8]. The under-sampled surgical resection specimen leads to inaccuracies in determining the final status of the surgical resection margins, in assessing the extent of disease, and in detecting multifocal disease or occult disease[8].

The emergence of near-infrared (NIR) fluorescence imaging is providing a new opportunity for real-time intraoperative imaging and assessment of surgical resection specimens [9, 10]. Several NIR fluorescence imaging systems are FDA approved and available for clinical use in humans: Novadaq SPY (Mississauga, ON), Photodynamic Eye (PDE, Hamamatsu, Hamamatsu City, Japan), Fluobeam (Fluoptics, Grenoble, France), FLARE imaging system (Frangioni Laboratory, Boston, MA) [11]. Such systems are generally rather large, bulky and cost prohibitive, making them relatively unavailable to surgeon in smaller community-based practice or in undeveloped countries. Recently, smaller fluorescence imaging systems have also been developed, such as a FluoSTIC system by Sylvain Gioux et al and a portable imaging system by Yukihiko Hiroshima et al[12–14]. These systems generally display intraoperative images on stand-alone monitor display screens, thus requiring the surgeon to divert attention away from the operative field and potentially resulting in the distraction of the surgeon during critical portions of the surgical procedure [15, 16]. To address these issues, one very viable solution is to display the real-time intraoperative imaging information on a wearable goggle device (i.e., Google Glass), so that the surgeon is able to obtain real-time intraoperative information and feedback without changing or diverting the field of view during the surgical procedure.

**Fig 1:**
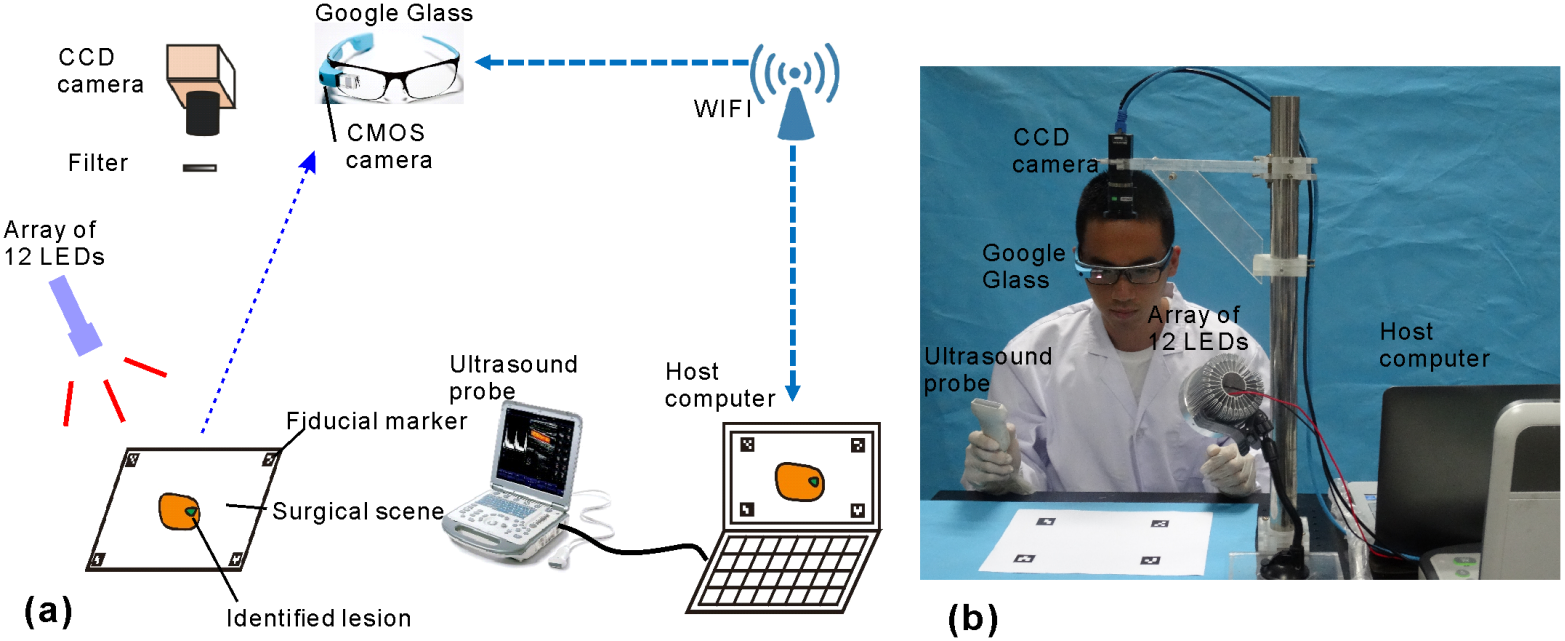
Prototype of the navigation system. Schematic depiction (a) and digital photograph (b) of the prototype wearable goggle navigation system.

We have previously developed a wearable goggle navigation system and demonstrated its feasibility for surgical navigation in an ex vivo tissue model [17]. Then we improved the navigation system and the primary utility was demonstrated in a single human subject during breast cancer surgery [18]. In the current paper, the design of the wearable goggle navigation system is improved and the algorithm and the navigation strategy have changed to facilitate dual-mode ultrasound and fluorescence imaging of the examined tissues. Fig 1 shows the schematic diagram of the improved system. It consists of a light emitting diode (LED) array for excitation light illumination, a stationary charge-coupled device (CCD) camera with a long pass filter for acquiring fluorescence images, a host computer for data processing, a Google Glass for displaying the fluorescence emission of ICG, and a clinical ultrasound system for providing necessary structural/anatomical information of the target lesion. During the simulated surgical procedure, the surgical scene image acquired by the Google Glass, the fluorescence image acquired by the CCD camera, and the ultrasound image acquired by the clinical ultrasound probe are all transferred to the host computer, processed, and sent back wirelessly to the Google Glass. With the help of the Google Glass, the surgeon is able to alternate back and forth between an ultrasound imaging mode and a fluorescence imaging mode, in order to obtain both positional laterality information and depth information about the sites of ICG uptake within the examined tissues and to guide the surgical resection procedure.

## 2. Methods

### 2.1 Hardware Design

The wearable goggle navigation system consists of a MV-VEM033SM monochromatic CCD camera (Micro vision, Xi’an, China) for fluorescence imaging, an array of 12 LEDs with central wavelength of 690 nm and overall power of 12 W (Sealand Opto Electronics Co., Shenzhen, China) for excitation light illumination, an M5 clinical ultrasound probe (Mindray, Shenzhen, China) for ultrasonography, and a Google Glass (Google Labs, Mountain View, CA) for acquiring and displaying surgical scene images. An FBH800-10 800 nm long pass filter (Thorlabs Inc. Newton, NJ) is used with the CCD camera to acquire fluorescence image at a resolution of 640 × 480 pixels and a frame rate of 30 frames per second (fps). The RGB image of the surgical scene is also acquired by a complementary metal-oxide semiconductor (CMOS) camera in the Google Glass at the resolution of 2528 × 1856 and the same frame rate. The acquired fluorescence and surgical scene images are transmitted to a host laptop computer, rescaled, processed, and transmitted back to the Google Glass display at a resolution of 640 × 360.

### 2.2 Surgical navigation strategy

**Fig 2:**
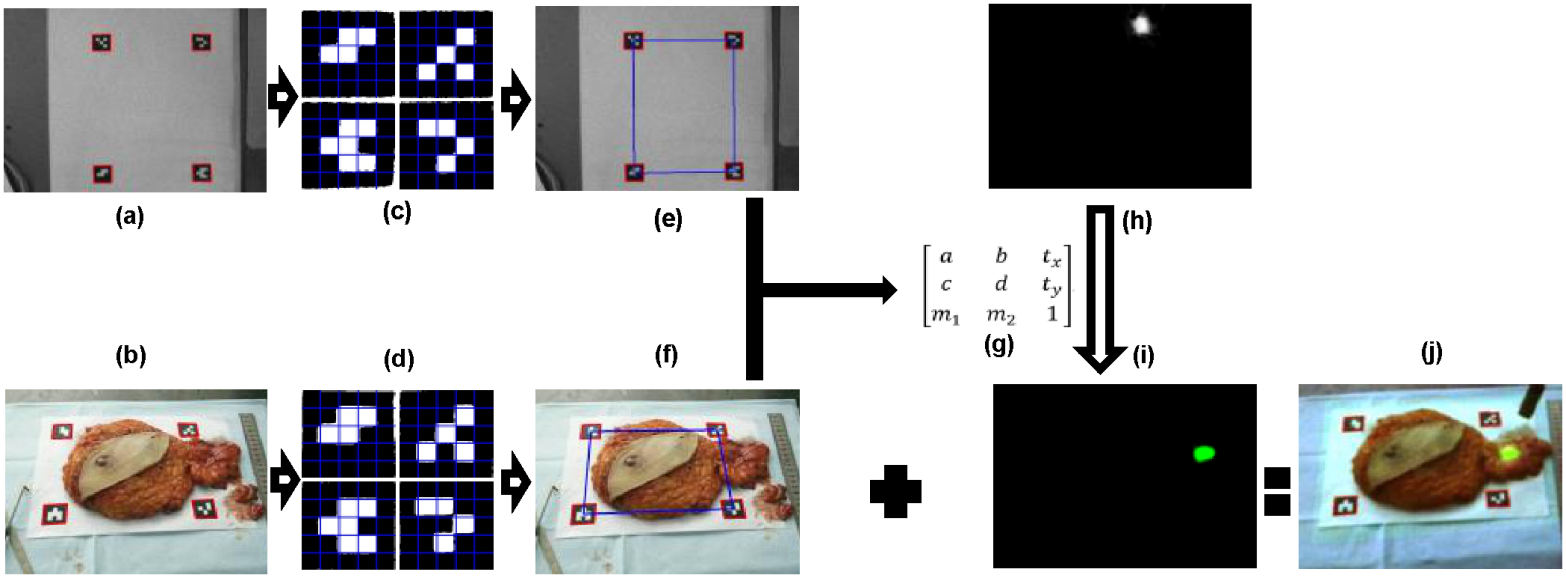
The surgical navigation strategy. (a)An image of the surgical scene (with four fiducial markers) is acquired by the stationary CCD camera without long pass filter. (b) RGB image of the surgical scene is acquired by the CMOS camera in the Google Glass. (c, d) 2-dimensional binary coded fiducial markers are detected and identified. (e, f)Centers of the fiducial markers are computed and set as the vertices of the fiducial quadrangle. (g) Transform matrix is generated. (h)Fluorescence image is acquired by the fixed calibrated CCD camera with a long pass filter. (i)The rectified fluorescence image after transformation using the transform matrix described above. (j) Image fusion of surgical scene after calibration, rectification, and co-registration.

The wearable goggle navigation system for surgical navigation is designed to support real-time display of the surgical scene in either fluorescence or ultrasound mode according to the surgeon’s needs. In the fluorescence mode, the fluorescence images acquired by the stationary CCD camera are seamlessly fused with the RGB images acquired by the Google Glass using four fiducial markers applied at four corners of the surgical field. In order to facilitate accurate co-registration, the cameras and the system are calibrated in advance using a calibration board and the fiducial markers. This standard photogrammetric technique provides internal parameters of both cameras, including focal length, principal points and lens distortions. Image co-registration across the devices is achieved following a step-by-step procedure as illustrated in Fig 2. First, the center coordinates of the four fiducial markers with unique identification numbers are computed using an image acquired in advance by the stationary CCD camera. Then, the fluorescence image acquired by the stationary CCD camera and the RGB image acquired by the Google Glass during the surgical procedure are co-registered using the fiducial markers. The fused image data is then sent back to the Google Glass for display. In the ultrasound mode, the clinical ultrasound system is connected to the host computer via a S-Video port so that the acquired ultrasound images of the surgical scene are transferred to the host computer, processed, and displayed in the Google Glass in real-time. All the above image processing procedure is implemented by C++ programming language that calls the OpenCV functions.

**Fig 3:**
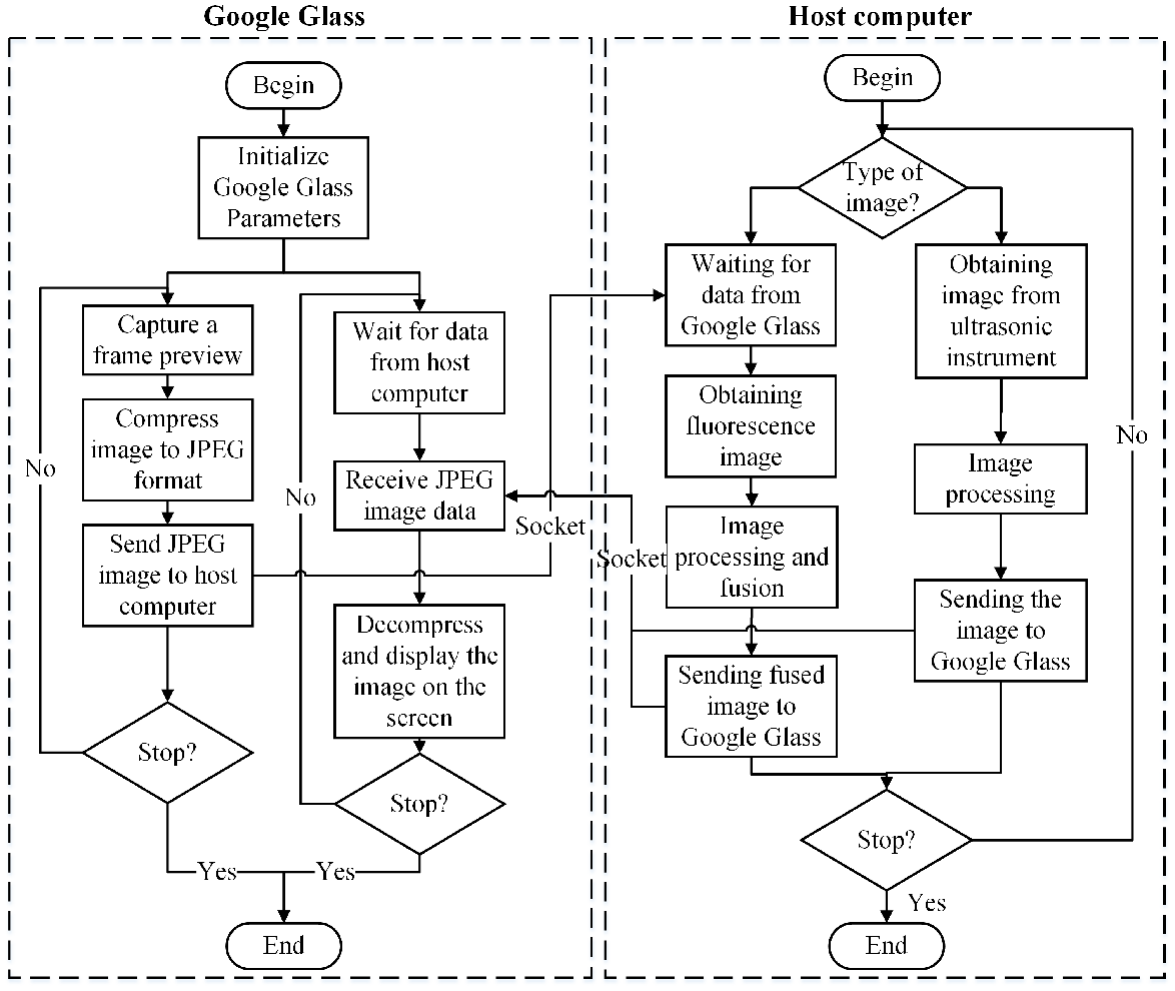
Program flow diagram for image acquisition, processing, transport and display on the Google Glass side and the host computer side of the surgical navigation strategy.

Fig 3 shows the flow chart of the programs running on the Google Glass side and the host computer side, respectively. The Google Glass carries out the following two tasks in parallel: (1) capturing an image through the CMOS camera on the Google Glass and sending it to the host computer, (2) receiving an image and displaying it on the Google Glass. The computer side displays the surgical scene in either fluorescence mode or ultrasound mode. In fluorescence mode, each frame of the RGB images acquired by the Google Glass and the corresponding frame of the fluorescence images acquired by the CCD camera are processed and the image fusion is sent back to the Google Glass for display. In ultrasound mode, each frame of the ultrasound images acquired by the clinical ultrasound probe is sent back and displayed on the Google Glass. Communication between the Google Glass and the host computer is implemented by a Transmission Control Protocol (TCP) socket through a Wi-Fi network. The Google Glass program is implemented by Android programming; while the computer program is implemented by Microsoft Visual C++.

### 2.3 Benchtop validation of the surgical navigation strategy in a tissue-simulating phantom

Technical feasibility of the proposed surgical navigation strategy is validated in a tissue-simulating phantom. The phantom is prepared by mixing 3% agar-agar gel, 7% glycerol, 0.004 g/mL TiO_2_, and distilled water to make a volume of 230mL approximately. The mixture is heated to 95°C and stirred for ~30 minutes on a magnetic stirrer. After cooling to 65°C, the mixture is poured in a plastic mold of 4*cm* × 4.5*cm* × 15*cm*. A cylindrical tube with the outer diameter of 1*cm* is placed 5*mm* below the top surface of the mold. After the mixture is completely cooled, the tube is carefully removed to form a cylindrical cavity. To prepare the lesion simulator, we mix 10% gelatin in distilled water to make a total volume of 10*mL*. The mixture is heated to 40°C for 40 seconds, stirred slowly for ~30–45 minutes while adding 0.04*g* of TiO_2_ gradually. After adding 0.08*mg* ICG and mixing for another 10 minutes, the mixture is poured into the cylindrical cavity of the phantom and cooled in a refrigerator for 2h. This process yields a solid phantom with an embedded fluorescence lesion simulator, which could easily be used to mimick the clinical scenario of fluorescence imaging of a site of tumor or fluorescence imaging of a sentinel lymph node (SLN).

### 2.4 Clinical validation of the surgical navigation strategy using sentinel lymph node (SLN) mapping and biopsy methodology

#### 2.4.1 Rationale of using the surgical navigation device for sentinel lymph node (SLN) mapping and biopsy

The SLN or SLNs are defined as the first lymph node or first lymph nodes to which cancer cells are most likely to drain from the site of the primary tumor. A SLN mapping and biopsy procedure is performed with the use of an agent which is injected into the tissues with the area of the primary tumor and which passively travels through the lymphatic channel draining from the area of the primary tumor and ultimately is taken up by the SLN or SLNs [19]. Resultantly, the SLN mapping and biopsy procedure will allow for the accurate identification, removal, and examination of the lymph nodes which are most likely to contain cancer cell which have lymphatically metastasized from the primary tumor site. If no lymph node metastases are found, patients can avoid unnecessary radical surgical removal of the contents of entire regional lymph node basins, and avoid the subsequent development of adverse long-term side effects, such as chronic pain and lymphedema.

#### 2.4.2 Rationale of using ICG as the localizing agent for sentinel lymph node (SLN) mapping and biopsy

Multiple agents and multiple injection routes can be used to successfully perform SLN mapping and biopsy [20, 21]. In contrast to radiolabeled agents, fluorescence agents can be used for intraoperative detection of SLN without the concerns and issues related to the handling and disposal of radioactive wastes. ICG is an FDA approved fluorescence enhancement agent for vascular and lymphatic imaging [22]. Considering that fluorescence imaging provides two-dimensional identification and mapping of the SLNs without depth information and with limited penetration depth, it is clinically advantageous to provide additional information regarding anatomic tissue structure and the depth of the SLNs located within areas of more thick tissues. Therefore, dual-mode imaging, accomplished by combining the use of fluorescence imaging with ICG and the use of ultrasound imaging (i.e., anatomic structural imaging) provides complimentary information for improved intraoperative guidance during SLN mapping and biopsy. The wearable goggle navigation system provides a real-time platform for allowing the surgeon to conveniently switch back and forth between a fluorescence mode and an ultrasound mode during the SLN mapping and biopsy procedure.

#### 2.4.3 Sentinel lymph node (SLN) mapping and biopsy methodology in an ex vivo human breast and axillary tissue specimen

**Fig 4:**
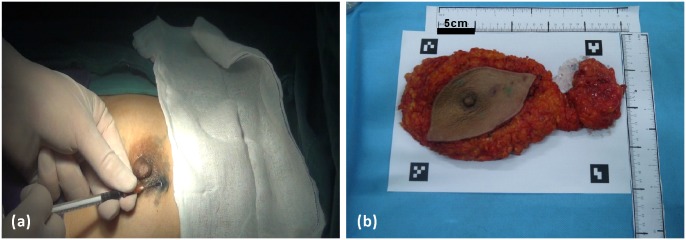
Ex vivo human breast and axillary tissue specimen. (a) Intradermal injection of ICG. (b) Modified radical mastectomy specimen, including axillary content.

The SLN mapping and biopsy methodology presented herein is demonstrated in a freshly excised human breast and axillary tissue specimen. The clinical protocol is in compliance with the Declaration of Helsinki and approved by the Institutional Review Board of the First Affiliated Hospital of Anhui Medical University (Protocol No: AF/SC-08/02.0). Written informed consent is provided by the patient. The individual in this manuscript also has given written informed consent (as outlined in PLOS consent form) to publish these case details. Due to the current regulatory restrictions of the First Affiliated Hospital of Anhui Medical University, in vivo validation of the proposed surgical navigation technique is very challenging. Therefore, ex vivo validation tests are designed to demonstrate the technical feasibility of the proposed surgical navigation strategy. The specific patient recruited for this study is already diagnosed with breast cancer and is scheduled for a standard surgical approach with a modified radical mastectomy. About 5 minutes prior to initiation of the surgical procedure, 1 mL ICG solution at a concentration of 0.6mg/mL (Dandong Yichuang Pharmaceutical Co., Ltd, China) is injected intradermally around the mammary areola of the right breast [23](Fig 4a). In order to help transportation to the lymphatic vessels, the injection area is then massaged with alcohol wipes for approximately 10–20 seconds [24–28]. During the modified radical mastectomy, the entire breast tissue and the axillary content tissues are surgically removed (Fig 4b). The excised tissue specimen is then transported to a separate designated area outside of the operating room for performance of the SLN mapping and biopsy methodology on this ex vivo human breast and axillary tissue specimen, which is carried out within 1–2 hours of the time of the surgical excision. With the guidance of the wearable goggle navigation system, SLNs and surrounding tissues are identified and excised from the ex vivo human breast and axillary tissue specimen. The SLNs and surrounding tissues undergo standard histological processing and histopathologic microscopic evaluation. Per standard protocol [19], SLNs are serially divided into 2mm thick portions, placed in cassettes, fixed in formalin, embedded in paraffin. Subsequently, 250*μm* thick microtome sections are cut, mounted on glass slides, stained by standard hematoxylin and eosin (H&E) staining methods, and microscopically examined. If no carcinoma cell is identified within a SLN on H&E evaluation, the SLN is serially sectioned at intervals of 5*μm* and re-examined by H&E and cytokeratin immunohistochemistry. The excised surrounding tissue without SLNs is microscopically examined by a standard H&E staining alone. All excised tissues are microscopically examined with a BX51 microscope (Olympus Corporation, Japan).

## 3. Results

### 3.1 Optical distortion for camera lens of Google Glass

**Fig 5:**
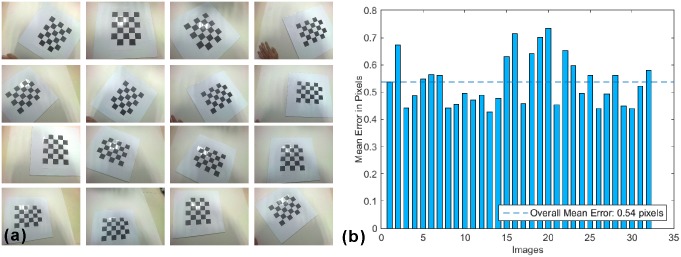
Checkerboard image acquisition and analysis. (a) The checkerboard images are acquired by the Google Glass at different orientations and different imaging distances. (b) The bar graph displays the mean reprojection errors for the acquired images, with an average error of around 0.54 pixels.

Considering that the Google Glass’s camera has a wide field of view, the induced non-uniform optical distortion has to be evaluated[29]. For this purpose, a 5 × 6 checkerboard with pixel size of 40 × 40 mm is prepared. The checkerboard is placed at different distances from 250 mm to 450 mm away from the Glass and at different orientations. As shown in Fig 5(a), the checkerboard images are acquired by the Glass at a resolution of 2528 × 1856. A MATLAB code is programmed to calculate the mean reprojection error of each image. According to Fig 5(b), the mean reprojection errors of all the acquired images are averaged about 0.54 pixels, corresponding to 0.09 mm in the scenario of breast cancer SLN resection where the surgical field is less than 420 × 297 mm. Considering that this level of reprojection error is much smaller than that required for SLN resection, no further calibration is applied for correcting the Glass induced optical distortion during surgical navigation.

### 3.2 Benchtop validation of the surgical navigation strategy in tissue-simulating phantoms

**Fig 6:**
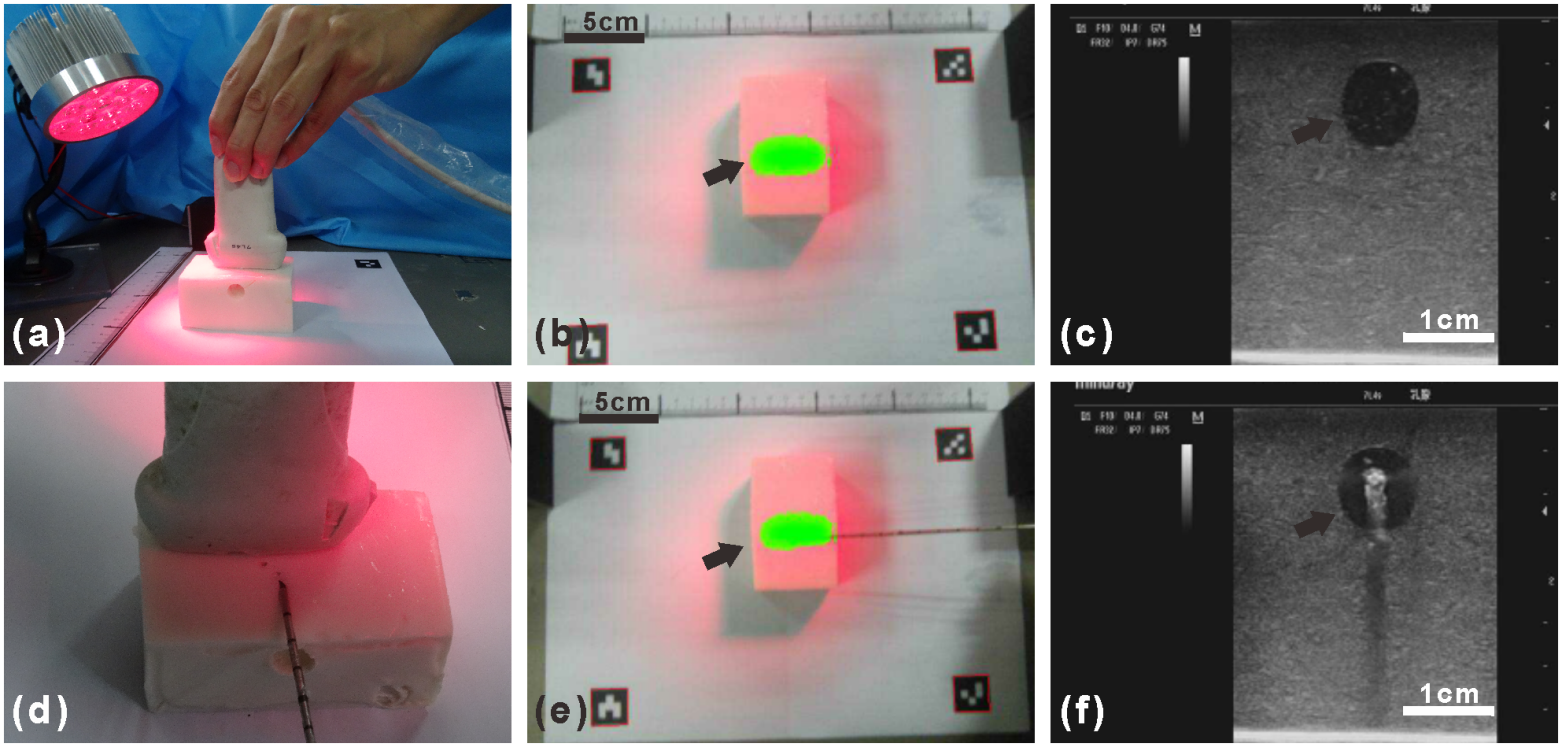
Ultrasound and fluorescence image-guided localization and core needle biopsy of a simulated tumor within a tissue-simulating phantom. (a) Detecting the simulated tumor within the tissue-simulated phantom with the LED light and ultrasound probe. (b)Fluorescence image of the simulated tumor show in green (arrow) within the tissue-simulated phantom. (c) Ultrasound image of the tissue-simulated phantom with the simulated tumor shown as the circular hypoechoic region (arrow). (d) The core needle biopsy device toward the simulated tumor. (e) The fluorescence image guided the advance of the core needle biopsy device toward the simulated tumor show in green (arrow). (f) The ultrasound image guided the advance of the core needle biopsy device toward the simulated tumor (arrow).

**Fig 7:**
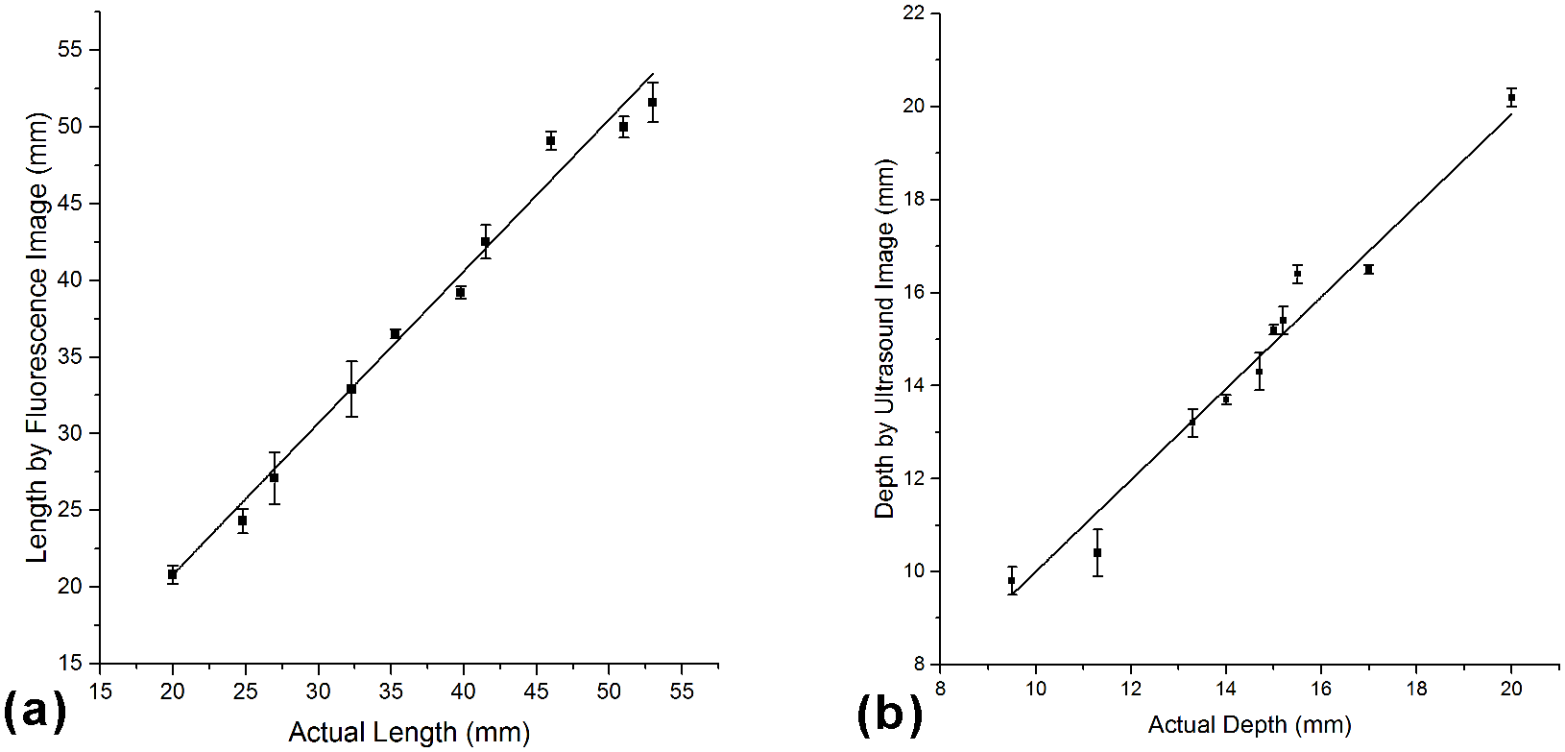
Accuracy of the navigation system in the lateral and the depth directions. (a)In the lateral direction, the length measured by fluorescence image is linearly correlated with the actual length (r = 0.9897). (b) In the depth direction, the depth measured by ultrasound image is linearly correlated with the actual depth (r = 0.9795).

Fig 6 shows experimental setup for localization and biopsy of a simulated tumor within the tissue-simulating phantom. A cylindrical insert filled with ICG is placed 5 mm below the surface of the tissue-simulating phantom to simulate ICG uptake within a tumor (Fig 6a). The Google Glass effectively projects the fluorescence image and the ultrasound image of the simulated tumor at a frame rate of 6 fps and 15 fps, respectively. The fluorescence image in Fig 6b shows the top view of the tissue-simulating phantom, with the simulated tumor shown in green. The ultrasound image in Fig 6c shows the cross-sectional view of the tissue-simulating phantom, with the simulated tumor shown as the circular hypoechoic region. By combining Fig 6b and 6c, one can easily localize the simulated tumor with accuracy within a three-dimensional space. In order to determine the localization accuracy of the navigation system, a series of phantoms are prepared by embedding the simulated tumor at different depth and length to the edge. In the lateral direction, fluorescence images taken by the system are analyzed by MATLAB to calculate the length between the center of the fluorescence part and the edge of the phantom. Fig 7a shows that the length measured by fluorescence images is linearly correlated with the actual length (Pearson correlation coefficient r = 0.9897).In the depth direction, ultrasound images are used to measure the depth from the center of the simulated tumor to the surface. The figures obtained by ultrasound images are linearly correlated with the actual depth (r = 0.9795, see Fig 7b). The localization accuracy is better than 3.1 mm in the lateral direction and better than 1 mm in the depth direction.

The fluorescence and the ultrasound images are used to guide the advance of the core needle biopsy device toward the simulated tumor. Fig 6e and 6f shows the fluorescence and the ultrasound images of the phantom where the core needle biopsy device effectively targets the simulated tumor. The simulated core needle biopsy procedure is carried out 30 times to compare the success rate for a single core needle puncture to hit the target in the following scenarios: (1) under Google Glass guidance of dual-mode imaging, (2) under Google Glass guidance of fluorescence imaging only, (3) without Google Glass guidance. The experimental results show that dual-mode Google Glass navigation guidance results in the highest biopsy success rate of 100% (10/10). In comparison, Google Glass guidance of fluorescence imaging only yields a moderate biopsy success rate of 70% (7/10), and that simulated biopsy without Google Glass guidance yields a biopsy success rate of 20% (2/10). This experiment implies that this navigation system can be potentially used in guided needle biopsy.

### 3.3 Dual-mode fluorescence and ultrasound imaging of a SLN in an ex vivo human breast and axillary tissue specimen

**Fig 8:**
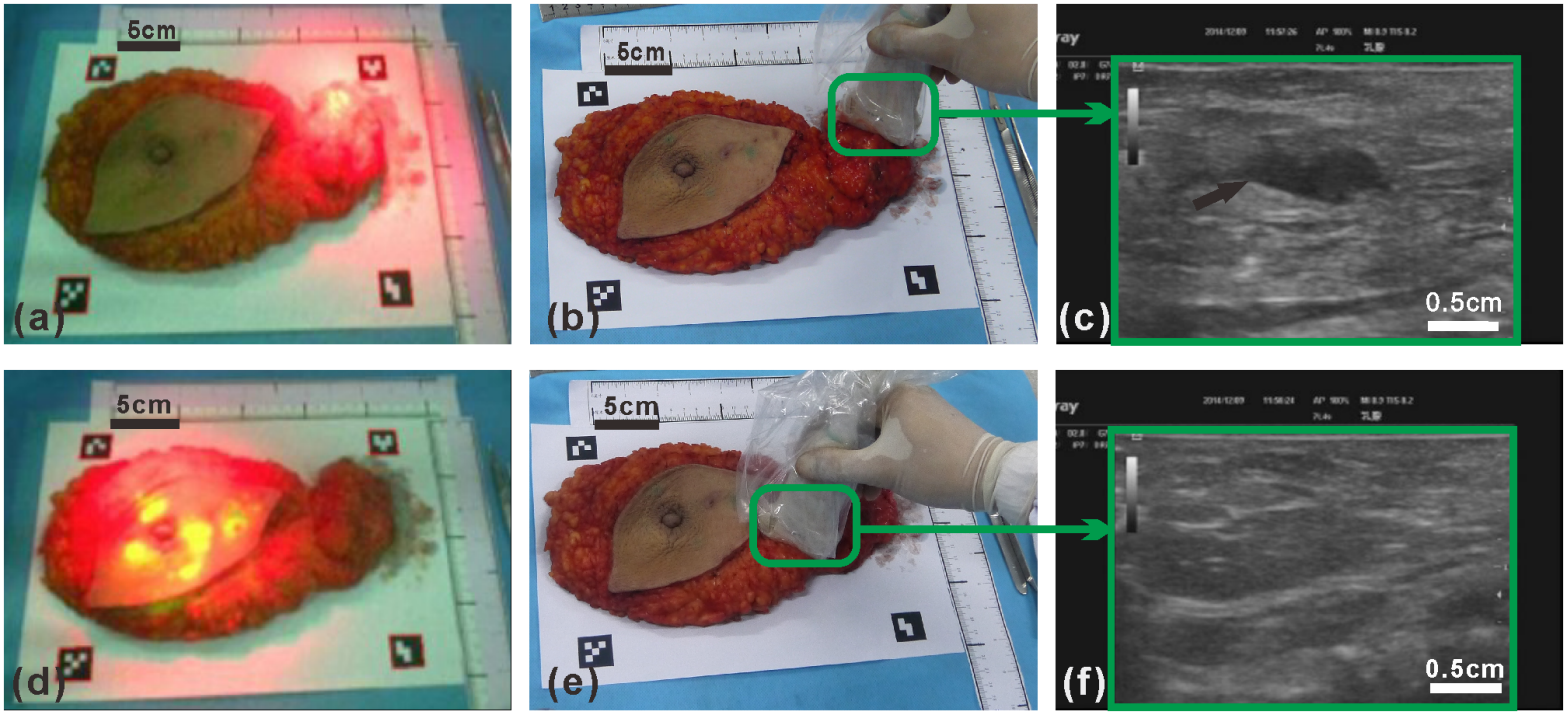
Fluorescence and ultrasound image-guided location of a SLN in 3 dimensions within the ex vivo human breast and axillary tissue specimen. (a) Fluorescence images of the modified radical mastectomy specimen with the attached axillary content with the LED light near the axillary content. (b, c) Ultrasound performed within the axillary region area of the modified radical mastectomy specimen showing a suspicious axillary lymph node (arrow). (d) Fluorescence images of the modified radical mastectomy specimen with the attached axillary content with the LED light near the nipple area. (e, f) Ultrasound performed within the lateral breast region of the modified radical mastectomy specimen showing the normal area of the breast tissue.

As shown in Fig 8, the ex vivo human breast and axillary tissue specimen is placed on a white board surrounded by four fiducial markers and illuminated by an array of LED lights. Through the wearable goggle navigation system, fluorescence emission is clearly visible in the axillary tissue region where the SLNs are visualized (Fig 8a) and in the periareolar region of the breast specimen where the ICG is injected (Fig 8c). When the ultrasound probe is placed over the location of the fluorescence-visualized SLN (Fig 8b), a hypoechoic ultrasound structure (arrow) is clearly visualized through the Google Glass, and the depth of the hypoechoic ultrasound structure is approximately 1 cm in its longest axis (Fig 8c). In contrast, as the ultrasound probe is moved to the area of tissue without fluorescence emission (Fig 8e), no definable hypoechoic ultrasound structures (Fig 8f) are detectable by the ultrasound probe. This experiment demonstrates the clinical usability of the wearable goggle device information and navigation system for fluorescence and ultrasound dual-mode imaging of SLNs.

### 3.4 Google Glass-guided SLN core needle biopsy in an ex vivo human breast and axillary tissue specimen

**Fig 9:**
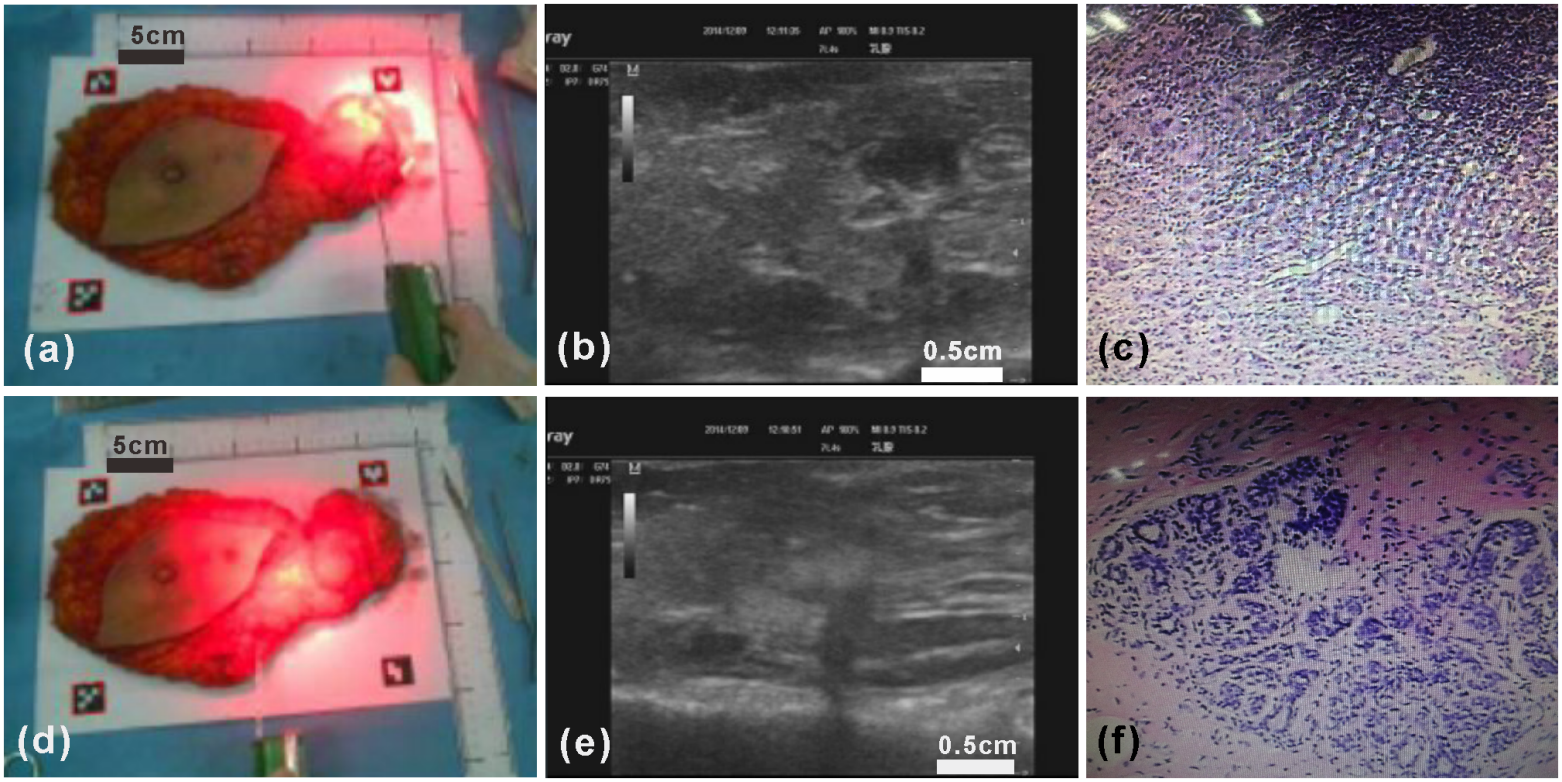
Core needle biopsy and microscopic histopathology analysis of an ICG-fluorescing SLN and non-fluorescing breast tissue from the ex vivo human breast and axillary tissue specimen using Google Glass guidance. (a-c) Ultrasound-guided core needle biopsy of an ICG-fluorescing SLN of the breast tissue specimen and microscopic histopathology analysis showing the presence of carcinoma. (d-f) Ultrasound-guided core needle biopsy of a non-fluorescing area of the breast tissue specimen and microscopic histopathology analysis showing normal breast tissue only.

In this experiment, the Google Glass navigation system is used to guide the ultrasound-guided core needle biopsy of an ICG-fluorescing SLN seen with the ex vivo human breast and axillary tissue specimen (Fig 9). During the biopsy procedure, the Google Glass display is switched between the fluorescence mode and the ultrasound mode, as based on the surgeon’s needs, in order to accurately achieve imaging and localization of the ICG-fluorescing SLN in a 3-dimensional fashion. Once the location of the ICG-fluorescing SLN is accurately determined to the satisfaction of the surgeon, a core needle biopsy device is used to harvest tissue core samples from the targeted ICG-fluorescing SLN. Microscopic histopathology analysis of the ICG-fluorescing SLN tissue core samples demonstrates the presence of carcinoma within the sampled lymph node tissue (Fig 9c). As the control experiment, an ultrasound-guided core needle biopsy is performed, in a similar fashion, to an area breast tissue within the lower outer aspect of the breast specimen where no fluorescence emission or hypoechoic ultrasound structures are visualized (Fig 9d and 9e). Microscopic histopathology analysis of the non-fluorescing area of the breast tissue specimen shows normal breast tissue only (Fig 9f). This experiment demonstrates that the Google Glass navigation system provided useful guidance for core needle biopsy of an ICG-fluorescing SLN.

### 3.5 Google Glass navigation system-guided excision of a SLN in an ex vivo human breast and axillary tissue specimen

**Fig 10:**
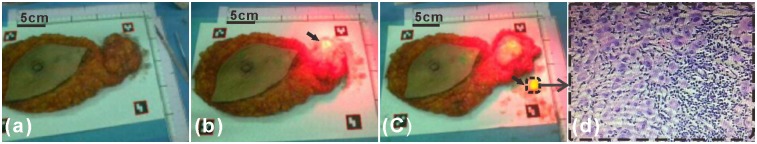
Fluorescence image-guided excision and microscopic histopathology analysis of an ICG-fluorescing SLN from the ex vivo human breast and axillary tissue specimen using the Google Glass system guidance. (a) Image of the ex vivo human breast and axillary tissue specimen without fluorescence illumination of the LED light. (b) SLN (arrow) visualized with the fluorescence images before attempted excision. (c) Image of SLN (arrow) localized and excised with the system guidance of the fluorescence imaging. (d) Microscopic histopathology analysis showed the presence of carcinoma within the excised lymph node.

In this experiment, the Google Glass navigation system is used to guide the excision of an ICG-fluorescing SLN within the ex vivo human breast and axillary tissue specimen (Fig 10). Without fluorescence imaging, there is no visible or tactile/palpable clue to allow for identification of any SLNs within the ex vivo human breast and axillary tissue specimen (Fig 10a). With fluorescence imaging, the ICG-fluorescing SLN is clearly visualized (Fig 10b). Using the wearable goggle navigation system, the ICG-fluorescing SLN is successfully localized and excised (Fig 10c). Microscopic histopathology analysis of the excised ICG-fluorescing SLN demonstrates the presence of carcinoma within the excised lymph node (Fig 10d). This experiment demonstrates that the Google Glass navigation system could be used to guide successful excision of ICG-fluorescing SLNs.

## 4. Discussion

This paper reports on the development and validation of a wearable goggle navigation system for dual-mode optical and ultrasound imaging and localization of sites of near-infrared emitting optical agent uptake. This Google Glass navigation system appears fully adaptable to the surgical management of breast cancer. As compared with the other fluorescence-guided imaging systems that have already been reported used in SLN mapping and biopsy, our system has two major advantages: (1) both fluorescence images and ultrasound images are acquired, in a dual fashion, allowing for improved 3-dimensional spatial localization of SLN candidates; and (2) fluorescence and ultrasound images are projected to a wearable goggle device (i.e., Google Glass) in such a fashion that does not interfere with the surgeon’s normal visualization of the surgical fields and does not divert or distract the surgeon’s attention during critical portions of the surgical procedure.

Although we use ICG-fluorescing SLN localization, biopsy, and excision as our clinical validation methodology for the demonstration of the clinical utility of our surgical navigation strategy, we strongly believe that the potential clinical applications of our wearable goggle navigation system for dual-mode optical and ultrasound imaging and localization of sites of near-infrared emitting optical agent uptake go far beyond the scope of just SLN mapping and biopsy. It is our belief that this Google Glass navigation system can be utilized and fully integrated by the surgeon into all aspects of oncologic surgery, including: (i) aiding in the complete resection of the primary tumor site(s)[30, 31], (ii) intraoperative identification of previously unrecognized multifocal disease or occult disease, (iii) intraoperative determination of completeness of surgical resection and (iv) determination of the final status of the surgical resection margins on the excised surgical specimen as well as within the resultant surgical excision cavity. Likewise, it is our belief that this Google Glass navigation system can be utilized and fully integrated by the pathologist into all aspects of the processing and evaluation of the excised surgical specimen within the pathology department. In addition to its applications in oncologic surgery, it is our belief that this the Google Glass navigation system also has many potential useful applications within the arena of wound healing and plastic surgery, especially since tissue oxygenation and blood perfusion can be evaluated in real-time[32]. Lastly, and which has already been evaluated by others, preoperatively acquired diagnostic body imaging, such as computed tomography, magnetic resonance imaging, positron emission tomography, and single-photon emission computerized tomography, can also be fused with intraoperative fluorescence imaging and displayed by the Google Glass navigation system to provide the improved surgical guidance[24].

The currently proposed Google Glass navigation system is still very preliminary in its development and refinement, and has several important limitations that need to be further evaluated and overcome. First, since ICG is not a tumor-specific contrast agent[33], the currently proposed Google Glass navigation system cannot be accurately utilized or relied upon to precisely delineate surgical resection margins. Therefore, to fully realize the potential clinical impact of the currently proposed Google Glass navigation system, it is important to ultimately integrate tumor-specific fluorescence targeting agents into the schema. Second, the currently proposed Google Glass navigation system has an overall imaging acquisition speed of 6–10 fps in fluorescence mode, which is insufficient to optimal track the normal speed of moments and maneuvers undertaken by a surgeon during any given surgical procedure. We have previously evaluated the “lagging” effect and found that our navigation system is able to track the surgical scene without significant lagging when the velocity of translational motion is less than 1m/min. However, significant lagging may occur when the surgeon moves much faster than this figure[17]. Therefore, it will ultimately be vitally important to optimize hardware and software technology aspects related to the speed of data acquisition and processing, in order to achieve real-time imaging guidance for the surgeon. Third, the existing design of the Google Glass utilized in the currently proposed Google Glass navigation system has not yet been optimized for surgical navigation applications. A preliminary clinical case demonstrating the clinical utility of the surgical navigation system has been discussed in our previous work. However, the fluorescence images displayed on the screen are not fused or co-registered with the surroundings[18]. Major drawbacks of the existing design of the Google Glass include low resolution of the screen display, short battery life, large heat dissipation in the Google Glass head set, inappropriate view point and long latency for data transmission [34]. It has been reported that the battery life of a Google Glass is typically 8.5–10 hours. However, our goggle navigation system has a much shorter battery life of about 1 hour, owing to the extra power consumption for display, data transport, and image processing. The working life of our goggle navigation system can be extended by using a portable power supply. Last but not least, when the navigation system is used to identify the targeted tissue visible by naked eyes, the augmented reality may induce inattentional blindness[35]. For this reason, the augmented reality function provided by our goggle navigation system is needed and helpful only when the targeted tissue cannot be well distinguished by naked eyes, such as in the case of SLNs mapping and resection. Therefore, further research and development of such a google navigation system requires careful consideration of the practical fields of application. In summary, it will ultimately be necessary to create a better platform of a wearable goggle device for optimized overall performance in surgical navigation.

It is the expectations that future upcoming work will focus on further engineering the design and clinical validation of this wearable goggle navigation system. The hardware and software configurations of the system will be optimized for more reliable performance, real-time display, and improved clinical utility. The limited clinical validation studies described in our current paper are simply based upon an ex vivo human breast and axillary tissue specimen. Our next step will be to demonstrate Google Glass-assisted sentinel lymph node mapping and biopsy procedure in vivo at the time of a breast cancer surgery. In addition to dual-mode fluorescence and ultrasound imaging, we are also exploring the potential for image fusion and intraoperative image display involving other modalities, such as computed tomography, magnetic resonance imaging, positron emission tomography, and single-photon emission computerized tomography [36–38]. In this regard, preoperatively acquired diagnostic body imaging information can be seamlessly integrated with intraoperative images to provide the improved guidance to the surgeon during surgical procedures.

## 5. Conclusions

We have developed and validated a wearable goggle navigation system for dual-mode optical and ultrasound imaging and localization of sites of near-infrared emitting optical agent uptake in both a tissue-simulating phantom and an ex vivo human breast and axillary tissue specimen. The results from our experiments support the contention that such a Google Glass navigation system can be potentially very useful and fully integrated by the surgeon for optimizing many aspect of oncologic surgery. Obviously, further engineering optimization and additional in vivo clinical validation work is necessary before such a surgical navigation system can be fully realized in the everyday clinical setting.
